# Development and substrate specificity screening of an *in vivo* biosensor for the detection of biomass derived aromatic chemical building blocks†
†Electronic supplementary information (ESI) available. See DOI: 10.1039/c6cc04559f



**DOI:** 10.1039/c6cc04559f

**Published:** 2016-08-25

**Authors:** Leopoldo F. M. Machado, Neil Dixon

**Affiliations:** a Manchester Institute of Biotechnology , University of Manchester , Manchester , UK . Email: neil.dixon@manchester.ac.uk

## Abstract

Measuring substrate and/or product concentration can create a major bottleneck for synthetic and biosynthetic processes. Here we report the development and substrate screening of a whole cell biosensor to detect biomass-derived aromatic chemical building blocks, supporting the use of sustainable feedstocks in the bulk and fine chemical industries.

## Abstract 2

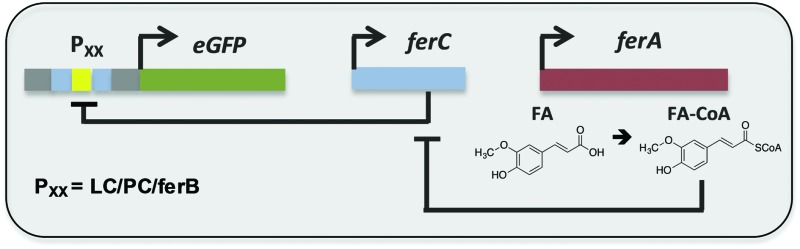
To facilitate the screening of chemical, enzymatic, and cellular processes to degrade and valorize plant biomass a whole cell biosensor was developed to detect lignin-derived substrates.

Valorization of biomass to create sustainable bio-synthetic routes to chemicals, plastics, monomers, waxes, fuel and energy is a central tenet of the move towards a circular bio-economy.^1^ An area of particular importance is the ability to valorize low value waste/by-products such as lignin. Degradation of lignin can release potential chemical building blocks that can be used as substrates for the production of high value chemicals, flavors, and fragrances.^2,3^ Production of high value chemicals from bio-based feedstocks can support the commercial feasibility of bio-fuels by utilizing a bio-refinery approach.^4–6^ Determination of substrate and/or product concentration can create a major bottleneck for chemo-enzymatic and whole-cell biosynthetic processes, as both off-line biochemical activity screening and analytical methods can be laborious. *In vivo* biosensors can provide a potential solution by enabling a real-time, intracellular read-out of the activity/phenotype.^7,8^ To facilitate the screening of chemical, enzymatic, and cellular processes to degrade and valorize plant biomass, we sought to develop a whole cell biosensor to detect lignin-derived substrates.

Lignin is a heterogeneous, polymeric, cross-linked material, mainly composed of monomers of *p*-coumaryl, coniferyl and sinapyl alcohols.^3^ Several thermo-chemical (kraft, sulfite),^9^ chemical (organosolv, alkaline hydrolysis),^10^ and thermo-pressure (steam explosion)^11^ based extraction methods have been used to degrade lignin.^1,4^ Enzymatic methods are currently expensive and require further optimization to be applied on a large-scale.^12,13^ Most well studied enzymatic processes are based on the use of isolated naturally occurring or recombinant fungal enzyme blends,^14,15^ however, bacterial lignin degrading enzymes have also been identified as promising alternatives.^16,17^ Degradation of lignin produces a mixture of phenylpropenoic acid monomers (*e.g. p*-coumaric, ferulic and caffeic acid). The ability to detect these lignin monomers would enable the optimization of enzymatic lignin degradation and valorization.

Here we report the development and substrate activity screening of an *in vivo E. coli* biosensor that permits the intracellular detection of substituted cinnamic acid scaffolds (*e.g.* ferulic acid). The system is based on the FerC repressor, a MarR-type repressor protein that binds to the DNA sequence upstream of the *ferB* gene (feruloyl-CoA hydratase) in *Sphingobium* sp. SYK-6.^18^ Previous *in vitro* studies identified that the interaction between FerC and two operator (IR1 and IR2) sequences upstream of the *ferB* gene is inhibited in the presence of the CoA-esters of coumaric, ferulic, and sinapic acid. In order to test a number of biosensor designs we performed promoter engineering to create variant promoter–operator sequences (Fig. S1, ESI†). Using the higher affinity IR2 operator site we generated three promoter–operator sequences, two chimeric phage promoters, (i) T7A1 promoter based (P_PC_) and (ii) lambda phage promoter based (P_LC_), and (iii) the wild-type *ferB* promoter/operator (P_*ferB*_). The relative constitutive expression levels from the three promoter variants were assessed by placing them upstream of the *eGFP* reporter gene (Fig. 1). The gene expression output was normalised to cell density (RFU/OD_600_), and plotted relative to the biosensor with the highest expression level (P_LC_). The P_*ferB*_ reporter produced the lowest relative expression level (∼20%), while the P_PC_ and P_LC_ reporters expressed intermediary (∼46%) and high expression levels in *E. coli* BL21, respectively (Fig. 2). The DNA sequences that encode the ferulic acid responsive repressor (FerC) and feruloyl CoA synthetase (FerA) were cloned and expressed from their constitutive promoters (Methods, ESI†). Expression of *ferC* resulted in repression of *eGFP* expression for all the biosensor designs. The addition of ferulic acid (FA) to the culture media led to de-repression of the biosensors and effective sensing of the intracellular presence of the substrate. The P_LC_ biosensor detected substrate concentrations over a 13-fold sensing range, with a ∼23-fold signal range. Fitting with a dose response curve indicates an EC_50_ of 20.9 ± 4.2 μM, saturation ≥100 μM of ferulic acid, and incomplete de-repression (∼85% of the P_LC_ reporter) (Fig. 2 and Table 1). The dose response curve for the P_PC_ biosensor indicates complete de-repression at ≥40 μM and an EC_50_ of 11.2 ± 1.1 μM (Fig. 2 and Table 1). The P_*ferB*_ biosensor was effectively de-repressed but had limited utility due to the small signal range. Considering the effects from the substrate perspective, FA has greater potency against the P_PC_ biosensor, but has a greater efficacy against the P_LC_ biosensor.

**Fig. 1:**
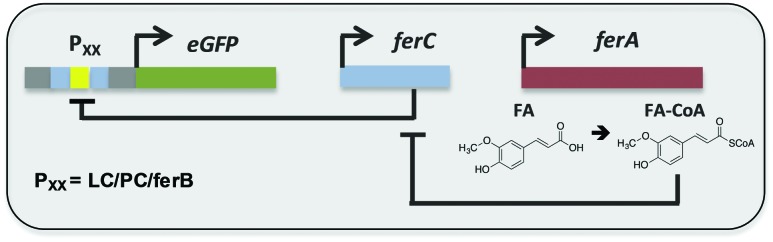
Biosensor design. Natural and engineered chimeric promoter–operators (P_*ferB*_, P_PC_, and P_LC_) were inserted upstream from the *eGFP* reporter gene. FerC binds to the promoter–operator sequence(s) repressing expression of the reporter gene. In the presence of FerA, ferulic acid (FA) is converted into feruloyl-CoA (FA-CoA), which in turn de-represses FerC and activates gene expression.

**Fig. 2:**
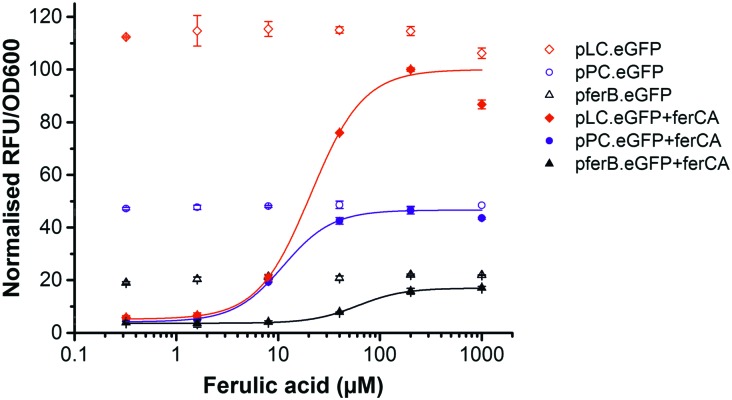
Biosensor performance. *eGFP* gene expression data in the absence (empty shapes) and presence of the *ferC* repressor (filled shapes), for the P_*ferB*_ (triangles), P_PC_ (circles), and P_LC_ (diamonds) biosensors in *E. coli* BL21. The fluorescent gene expression normalised to cell density (RFU/OD_600_) was expressed relative to the P_LC_ biosensor, and the dose response curves were fitted to increasing concentrations of ferulic acid.

**Table 1:** Signal range (max/min) and EC_50_ values from the fitted dose response curve for the three biosensor systems

Promoter	Signal range	EC_50_ (μM)
P_LC_	22.8	20.9 ± 4.2
P_PC_	10.4	11.2 ± 1.1
P_*ferB*_	5.0	60.5 ± 2.3

In order to validate the requirement of *ferA* and to provide *in vivo* validation of the previous *in vitro* observations,^18^ we created a *ferA* knock-out (AKO). This allowed us to confirm *in vivo* that the active substrate is indeed the coenzyme A (CoA) ester (Methods, ESI†). These strains lacked the de-repression phenotype upon addition of FA to growth media (Fig. S2, ESI†), confirming the essentiality of *ferA*, the FA-CoA ester as the ferC substrate, and the associated functional de-repression mechanism.^19^ We next explored the dependency of the biosensor performance upon the host *E. coli* strain used by testing the system in an *E. coli* K strain (DH10B). The absolute expression levels were lower in the K strain (Fig. S3 and Table S1, ESI†), however, the P_LC_ biosensor again produced the greatest relative signal range (∼19-fold), displayed an EC_50_ of 22.8 ± 5.2 μM, and a 13-fold sensing range (EC_90_/EC_10_) (Fig. S3 and Table S1, ESI†). As for the B strain (Fig. 2) incomplete de-repression was observed for the P_LC_ biosensor, whilst complete de-repression was again observed for both the P_PC_ and P_*ferB*_ biosensors. However, due to the enhanced signal outputs of the B strain and the P_LC_ biosensor it was decided that this was the most effective combination and was used for subsequent activity screening.

In order to assess the substrate specificity of the ferulic acid biosensor, we selected the P_LC_ biosensor for further screening and tested its activity against 58 structural analogues (Table S3, ESI†). Five substrate analogues were identified from the screening, which had high output signals, and hence good efficacy (>70%) against the biosensor (Fig. 3A) and displayed potencies with EC_50_ values ranging from 15 to 315 μM (Table 2). Based on the dose response curves, structure–activity relationships can be observed. Maximal potency requires a *para*-substituted phenyl ring with a hydrogen-bond donor (*p*-coumaric acid (**2**)), and a *meta*-methoxy substituent is also tolerated (ferulic acid (**1**)). Replacement of the hydroxyl substituent with an amino group (3-(4-aminophenyl)-2-propenoic acid (**3**)) results in loss of potency. Whereas, both the regioisomer of ferulic acid (3-hydroxy-4-methoxycinnamic acid (**5**)), and an additional *meta*-methoxy substituent on the phenyl ring (sinapic acid (**4**)), result in a more significant loss of potency (EC_50_ > 200 μM). The fitting of the dose response curves also indicates an extension of the sensing range for the different substrates. Ferulic acid is sensed over a 13-fold range, whereas sinapic acid presents the broadest predicted range (225-fold) (Table S2, ESI†). The next selection of substrates (**6–10**) (Fig. 3B) display a moderate signal (60–40%). Of these five substrates, one analogue (3,4-dihydroxy-5-methoxycinnamic acid (**6**)) displayed an EC_50_ of 746.5 μM with an extensive predicted sensing range (485-fold). The remaining moderately inducing substrates (2,4-dihydroxycinnamic acid (**7**), 3,4-dimethoxycinnamic acid (**8**), caffeic acid (**9**) and 4-nitrocinnamic acid (**10**)) displayed EC_50_ values ranging from 800 to 1600 μM (Table 2). The three remaining active substrates (3,4,5-trimethoxycinnamic acid (**11**), 3-(4-hydroxy-3-methoxyphenyl)propionic acid (**12**), and 3-methoxycinnamic acid (**13**)) displayed low signals (<40%) and low potencies (>2000 μM). The remaining 45 analogues displayed no observable activity against the FerC biosensor (Fig. S4 and Table S3, ESI†).

**Fig. 3:**
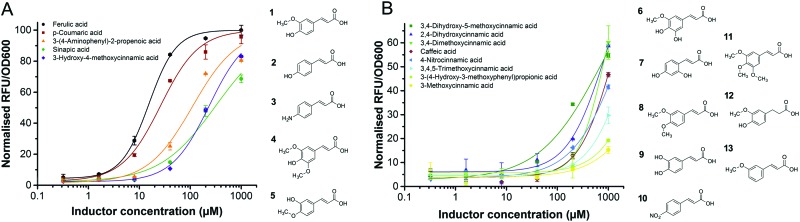
Biosensor responsive compounds. Dose response curves for the different compounds using the P_LC_ biosensor system in *E. coli* BL21 and their respective molecular structures. The fluorescent gene expression normalised to cell density (RFU/OD_600_) was expressed relative to the P_LC_ biosensor response curve with ferulic acid. Five compounds (**1–5**) generated high levels of expression with efficacy superior to 70% (A). Eight compounds generated moderate levels of expression with efficacies ranging from 10% to 55% (**6–13**) (B). * Compound numbers with respective names are described in Table 2 and Table S3 (ESI†).

**Table 2:** Signal range (max/min) and EC_50_ values from a dose response curve fitting for all responsive compounds tested

	Tested compounds	Induction	Signal range	EC_50_ (μM)
**1**	*trans*-Ferulic acid	H	26.2	15.3 ± 0.9
**2**	*p*-Coumaric acid	H	25.0	26.1 ± 3.8
**3**	3-(4-Aminophenyl)-2-propenoic acid	H	28.1	110.2 ± 25.7
**4**	Sinapic acid	H	15.4	314.4 ± 55.8
**5**	3-Hydroxy-4-methoxycinnamic acid	H	33.5	234.3 ± 15.4
**6**	3,4-Dihydroxy-5-methoxycinnamic acid	M	14.8	746.5 ± 117.7
**7**	2,4-Dihydroxycinnamic acid	M	9.6	823.4 ± 45.6
**8**	3,4-Dimethoxycinnamic acid	M	11.4	825.0 ± 13.2
**9**	Caffeic acid	M	11.2	1176.4 ± 88.5
**10**	4-Nitrocinnamic acid	M	9.5	1564.5 ± 96.8
**11**	3,4,5-Trimethoxycinnamic acid	L	6.7	2364.9 ± 264.9
**12**	3-(4-Hydroxy-3-methoxyphenyl)propionic acid	L	3.3	4687.1 ± 1189.6
**13**	3-Methoxycinnamic acid	L	4.8	9251.1 ± 11084.4

Analysis of the structure–activity relationships indicates that modest phenyl substituent changes can result in dramatic potency changes, for example an additional methyl group between 3,4,5-trimethoxycinnamic acid (**11**) and sinapic acid (**4**) results in an 8-fold change in potency. Similarly it can be observed that the substrate 3,4-dihyroxy-5-methoxycinnamic acid (**6**) is closely related to sinapic acid (**4**), and displays a slightly reduced potency (2.5-fold), whereas caffeic acid (**9**) displays a 55-fold loss in potency relative to the closely related ferulic acid (**1**). The low activity observed for 3-(4-hydroxy-3-methoxyphenyl)propionic acid (**12**) compared to ferulic acid (**1**) demonstrates the importance of the α–β unsaturated functionality. A number of observations can be made for the analogues that displayed no activity (Fig. S4 and Table S3, ESI†). Most interestingly, the un-substituted cinnamic acid (**14**) displayed no activity; in addition, the phenyl ring could not be exchanged for any other aromatic ring system (**23–28**); the importance of the carboxylic acid was confirmed by a lack of activity when removed (**30–34**); the distance between the phenyl ring and carboxylic acid was confirmed as essential (**39–46**) and the necessity of the α–β unsaturation for activity was confirmed (**16–22**). Finally, phenyl rings substituted with electron withdrawing halide groups (**50–54**), or electron donating methyl groups (4-methylcinnamic acid (**29**)) were also devoid of activity.‡
‡All assays were performed by addition of the substrates/supernatants directly to a culture of the biosensor containing *E. coli* strain(s), freshly grown to the appropriate cell density (OD 0.6). *eGFP* expression was monitored after three hours of growth/induction at 37 °C with shaking (1000 rpm). Cells were centrifuged, washed and re-suspended with PBS buffer. The expression output was then analyzed by monitoring the fluorescence normalised to cell density (RFU/OD_600_) in a multimode plate reader (Methods, ESI†).


To demonstrate the utility of the biosensor in both a practical application and against complex substrate mixtures, we screened for biosensor activity following enzyme treatment against a number of different biomass sources. In total, we used the biosensor to assess the activity of three feruloyl esterases (EC 3.1.1.73, CAZy, CE1) against three different biomass sources (Fig. 4). After enzyme treatment with *CE1-3* (from *C. thermocellum* DSM 1313) against wheat flour biomass, the biosensor screening confirmed efficient release of ferulic acid and/or closely related analogues, while treatment with enzymes *CE1-1* (from *A. cellulolyticus* CD2) and *CE1-2* (from *C. thermocellum*) resulted in reduced activity indicating only partial release. A similar relative activity profile was observed for the 3 enzymes against micronized oat husk biomass, however the total signal was reduced (>50%), suggesting lower levels of enzymatic release from this source. Thirdly, no activity was detected against kraft lignin biomass. The observed lower level and lack of activity may be due to the more recalcitrant nature of the particular biomass source or, in the case of kraft lignin, can be associated with the chemical pre-treatment process.^9^

**Fig. 4:**
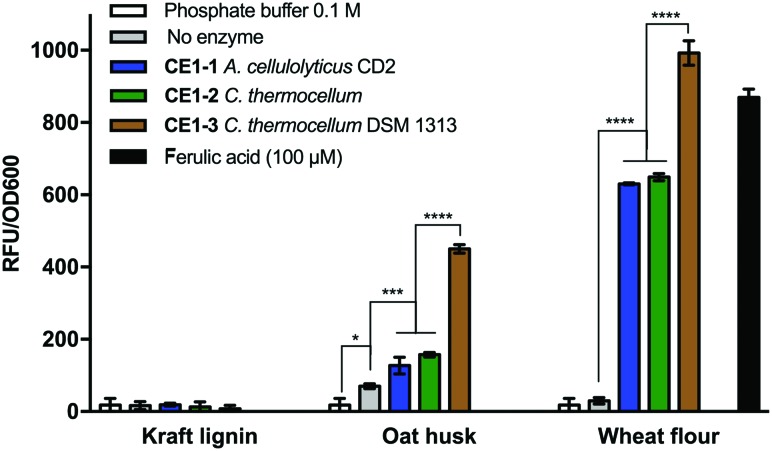
Biomass/lignin degradation screening with the P_LC_ biosensor system in *E. coli* BL21. Different lignin sources were submitted to treatment with three feruloyl esterase enzymes (CE1) or in the absence of enzyme and the supernatants were tested with the P_LC_ biosensor. The relative fluorescence to cell density (RFU/OD_600_) is shown for the feedstock treatments, with the phosphate buffer alone or with ferulic acid at 100 mM. (* *P* < 0.05, *** *P* < 0.001, **** *P* < 0.0001 analyzed by one-way ANOVA followed by Tukey's multiple comparison test).

In conclusion, the developed biosensor is able to detect 13 substituted cinnamic acid based compounds. The ferulic acid substrate is detected over a 13-fold sensitivity range, with >25-fold signal read-out range, and four other compounds display similar efficacy. The defined substrate specificity of this biosensor will enable its use in the identification and optimization of chemical and enzymatic processes. For example, processes which enable the de-polymerization of lignin and the release of chemical building blocks, in addition to processes which use these chemical building blocks as substrates for the production of high value chemicals including vanillin and flavonoids.^2,6,20^ Further applications include use of the biosensor in screening for the production of value-added compounds, and for substrate/product transport across biological membranes, which we are actively pursuing. This combination of applications will support the chemical using industries to source chemical building blocks from alternative sustainable bio-based feedstocks.

This research was supported by the Biotechnology and Biological Sciences Research Council (BBSRC) grants [BB/L026244/1]. ND holds a BBSRC David Phillips Fellowship [BB/K014773/1]. LM is supported *via* Science without Borders/Ciência sem fronteiras scheme from the CNPq, Brazil (233608/2014-1). We would like to thank Prof. David Leys’ group for use of their cinnamic acid compound library, Prof. Andrew Doig for useful discussions regarding data fitting, Dr Fabio Squina for providing the wheat flour biomass, and Phil Metcalfe (BioPower Technologies Ltd) for provding the oak husk biomass.
