# Supplementary material for: Development and substrate specificity screening of an in vivo biosensor for the detection of biomass derived aromatic chemical building blocks
Source: Chem Commun (Camb). 2016 Aug 25;52(76):11402–5. doi: 10.1039/c6cc04559f (PMC5048394; doi:10.1039/c6cc04559f)
Supplement: Supplementary file 1 [file CC-052-C6CC04559F-s001.pdf]

Electronic Supplementary Material (ESI) for ChemComm.
This journal is © The Royal Society of Chemistry 2016

Electronic Supplementary Information for

Development and substrate specificity screening of an *in vivo* biosensor for the detection of biomass derived aromatic chemical building blocks

Leopoldo F.M. Machado[1,], Neil Dixon[1,*]

*neil.dixon@manchester.ac.uk

[1]Manchester Institute of Biotechnology, University of Manchester, Manchester, United Kingdom

Material and Methods

Figure S1: FerB promoter region in Shingobium sp. strain SYK-6 and chimeric promoters designs

Figure S2: FerA Knockout system test

Figure S3: Reporters and Biosensor systems tested in E. coli DH10B

Figure S4: Biosensor non-responsive compounds

Table S1. Signal range (max/min) and fitted dose response curve data for the three biosensor systems in BL 21 and Top10F' strains

Table S2. Signal range (max/min) and fitted dose response curve data for all responsive compounds tested.

Table S3. CAS number and molecular structure of all compounds screened

Material and Methods

All cells were grown in LB medium (0.5% yeast extract, 0.5% NaCl, 1.0% Bactotryptone). A BMG CLARIOstar Microplate Reader was used to measure the GFP fluorescence and OD_{600} for intact cells.

Strains, Plasmids, Kits, Compounds.

DH10B (Top10F', Life Technologies), BL21 (Agilent), pET44-eGFP, p15 and synthetic sequences for: P_{LC}, P_{PC}, P_{ferBA}, FerA, FerC (*GeneArt*), Ampicillin (*Sigma*), Chloramphenicol (Sigma). Screened compounds: Trans-Ferulic acid, p-Coumaric acid, 3-(4-Aminophenyl)-2-propenoic acid, Sinapic acid, 3-Hydroxy-4-methoxycinnamic acid, 3,4-Dihydroxy-5-methoxycinnamic acid, 2,4-Dihydroxycinnamic acid, 3,4-Dimethoxycinnamic acid, Caffeic acid, 4-Nitrocinnamic acid, 3,4,5-Trimethoxycinnamic acid, 3-(4-Hydroxy-3-methoxyphenyl) propionic acid, 3-Methoxycinnamic acid, Cinnamic acid, 2-Hydroxycinnamic acid, 4-Methylcinnamic acid, a-Methylcinnamic acid, α-Fluorocinnamic acid, Phenylpropiolic acid, Sodium phenylpyruvate, L-Tyrosine, 3,4-Dihydroxy-L-phenylalanine (L-DOPA), 3-(2-Furyl)acrylic acid, 3-(2-Thienyl)acrylic acid, 4-Imidazoleacrylic acid, trans-3-Indoleacrylic acid, Cinnamamide, α-Acetamidocinnamic acid, Methyl cinnamate, 3-Phenylpropionic acid, 3-(3,4-Dihydroxyphenil)propionic acid, 3-(4-Hydroxyphenyl)propionic acid, 3-(3-hydroxy-4-methoxyphenyl)propionic acid, Syringic acid, Gallic acid, Benzoic acid, 3,4-Dihydroxybenzoic acid, Vanillic acid, 4-Hydroxy-3-methylbenzoic acid, 4-Hydroxybenzoic acid, Terephthalic acid, 2,5-Furandicarboxylic acid, 2,5-Thiophenedicarboxylic acid, Furoic acid, 4-Chlorocinnamic acid, 4-Fluorocinnamic acid, 4-Bromocinnamic acid, 1,2,3,4,5-Pentafluorocinnamic acid, trans-4-(Trifluoromethyl)cinnamic acid, trans,trans-Muconic acid, cis,cis-Muconic acid, Potassium sorbate, 2,4,6-Octatrienoic acid (*Sigma*); 3-(2-Naphthyl)acrylic acid, 3-(1-Napthyl)acrylic acid**,** 4-Vinylphenol**,** 2-Methoxy-4-vinylphenol (*Alfa Easer*); Methyl ferulate (*Fluorochem*).

Biomass sources and Enzymes.

Kraft lignin (*Sigma*); Wheat flour (arabinoxylan, insoluble) (*Megazyme*); Micronized oat husk fibre (kindly provided by *Biopower Technologies Limited)*. Recombinant feruloyl esterases (EC 3.1.1.73, CAZy CE1) from *Acetivibrio cellulolyticus* CD2 (**CE1-1**), *Clostridium thermocellum* (**CE1-2**) and *Clostridium thermocellum* DSM 1313 (**CE1-3**) (*Prozomix*).

Vector Engineering

The sequences containing the $P_{LC,}$ P_{PC} and P_{ferB} promoters, a RBS, and a Hexa-Histidine tag, flanked by *SphI/NdeI* were synthetized (GeneART, ThermoFisher). The sequences were enzyme restricted with *SphI/NdeI* and cloned in pET44eGFP,[2] upstream to the eGFP gene, replacing the T7 promoter region generating respectively, the pET44P_{LC}eGFP, pET44P_{PC}eGFP and pET44P_{ferB}eGFP vectors.

The FerCA DNA sequence containing the *ferA* and *ferC* genes, individually flanked by a PLacI promoter and an rrn_B1 terminator, was synthetized by GeneART™ (ThermoFisher). The construct was cloned in a p15 plasmid flanked by *NaeI/KasI* restriction sites generating the p15FerCA vector. The *ferA* gene was removed by restriction digestion of two *XbaI* flanking sites. The remaining backbone with *ferC* was re-circularized to originate the p15FerC vector, and the obtained plasmid was sequenced to confirm identity.

Biosensors Performance and Screening Methods

Reporter controls and the biosensor systems $P_{LC,}$ P_{PC} and P_{ferB} were respectively generated by transformation of pET44P_{LC}eGFP, pET44P_{PC}eGFP and pET44P_{FerB}eGFP vectors alone or with p15FerCA in BL21 and DH10B chemically competent *E. coli* cells. A single colony of each system was grown in LB media supplemented with antibiotics at 37 °C with shaking at 180rpm for 16 hours. Cultures were diluted (1:100) in fresh LB media with appropriate antibiotics, re-incubated at 37 °C with shaking until OD ~0.6 and transferred (450 µL) to multi-well plates containing Ferulic acid at concentrations of 0.32 µM, 1.6 µM, 8.0 µM, 40 µM, 200 µM and 1000 µM. Induction plates were incubated at 37 °C, with shaking at 1000 RPM (Stuart microtitre plate shaker incubator) for 3 hours. Cells were harvested by centrifugation, washed twice and re-suspended with PBS buffer. Expression output was analyzed by monitoring the fluorescence normalised to cell density (RFU/OD) in a multimode plate reader.

FerA knockout systems of each promoter were generated by transformation of pET44P_{LC}eGFP and pET44P_{ferB}eGFP with the p15FerC vector in BL21 *E. coli* cells. The AKO and the biosensor cells were grown and the FA induction assay was repeated.

Compounds for screening were selected using cinnamic acid as a reference structure. The 58 selected compounds were tested with the P_{LC} biosensor in *E. coli* BL21 cells. The substrate screening assays were performed using concentrations ranging from 0.32 µM to 1000 µM, as described for Ferulic acid. All experimental data are the mean of at least two biological replicates.

Biomass enzymatic degradation and screening

The three biomass sources kraft lignin, wheat flour and micronized oat husk were individually mixed with each feruloyl esterase (CE1) enzyme in a 200:1 weight ratio (10 mg : 0.1 mg). One control, without enzyme, was made for each source. Phosphate buffer (0.1 M, pH 6.5) was added to 500 µL final volume and the tubes were incubated at 60 ºC with shaking at 1000 RPM (ThermoMixer *Epperdorf*) for 12 hours. Enzymatic reactions were centrifuged at 13,800 G for 15 minutes and the supernatants were collected.

Screening to detect released phenolic compounds was performed with the pLC biosensor in *E. coli* BL21. 50 µL of supernatant, phosphate buffer or FA (100mM) were mixed with 200 µL of culture at OD 0.6 in triplicates and the screening was followed as described for the FA induction assay.

Data Processing and Curve Fitting

Biosensor signal output (eGFP expression) was measured as Relative Fluorescence Units (RFU) and normalised to cell density (OD_{600}). The background auto-fluorescence of E. *coli* was subtracted from RFU/OD and was normalised (%) to the pLC biosensor response curve to ferulic acid for each experiment. The normalised data was plotted and fitted with a dose-response curve using the Levenberg Marquardt logistic growth/sigmoidal algorithm, using the Origin 2015 (OriginLab, Northampton MA USA) program. The biomass screening data was plotted and the statistic analysis was made using one-way ANOVA followed by Tukey's multiple comparisons test, with the GraphPad Prism 7.00 (GraphPad, La Jolla CA USA).

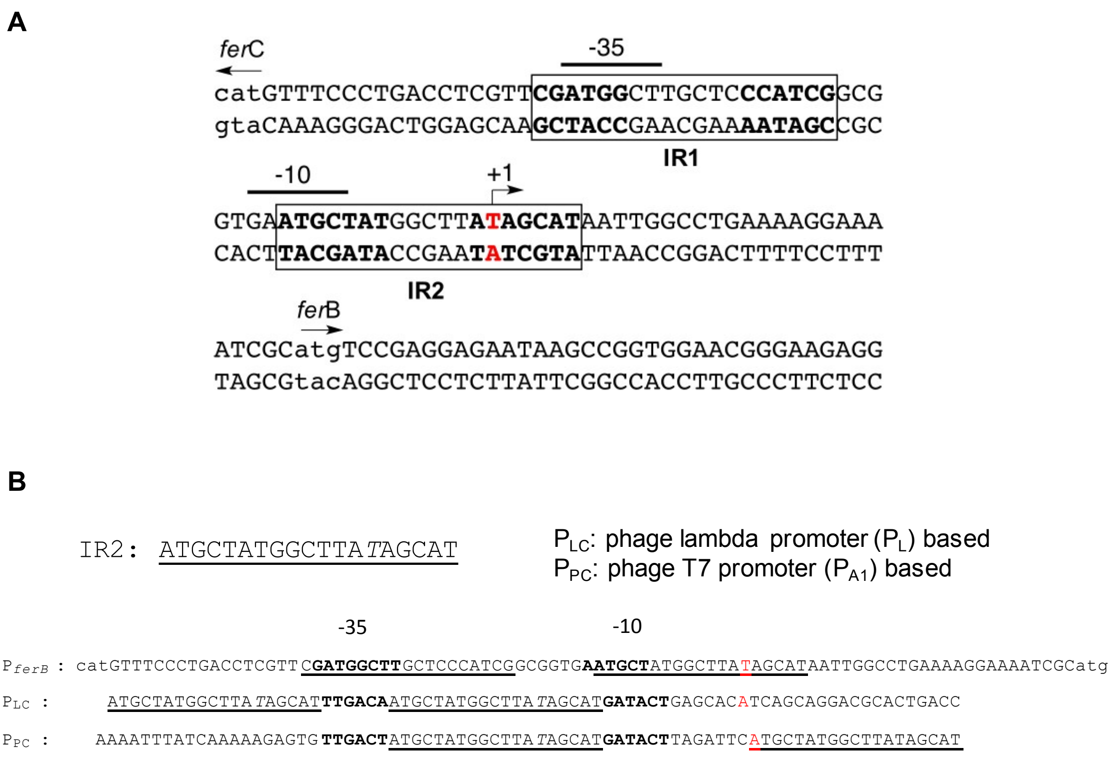

Figure S1: FerB promoter region in *Shingobium sp.* strain SYK-6 and chimeric promoters designs. The intergenic region between ferC (reverse) and ferB (forward) in *Sphingobium sp.* SYK-6 is shown. The Inverted Repeat sequences (IR1 and IR2) associated with FerC interaction inside the FerB promoter region, as described previously described[1], are highlighted (**A)**. Three promoter designs were constructed. One promoter based in the *Sphingobium* native promoter (P_{ferB}) and two chimeric promoters based in the region IR2 and the phage lambda promoter (P_{LC}) or the phage T7A1 promoter (P_{PC}) (**B**).

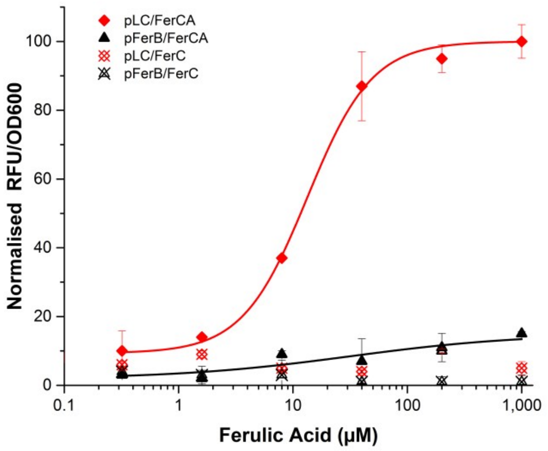

Figure S2: FerA Knockout system test. Ferulic acid induced expression using the Biosensor systems P_{LC} and P_{ferB} (filled shapes) and the absence of expression using the respective FerA Knockout (AKO) systems (crossed shapes) in *E. coli* BL21. Fluorescence normalised to cell density (RFU/OD) was expressed relative to the P_{LC} biosensor.

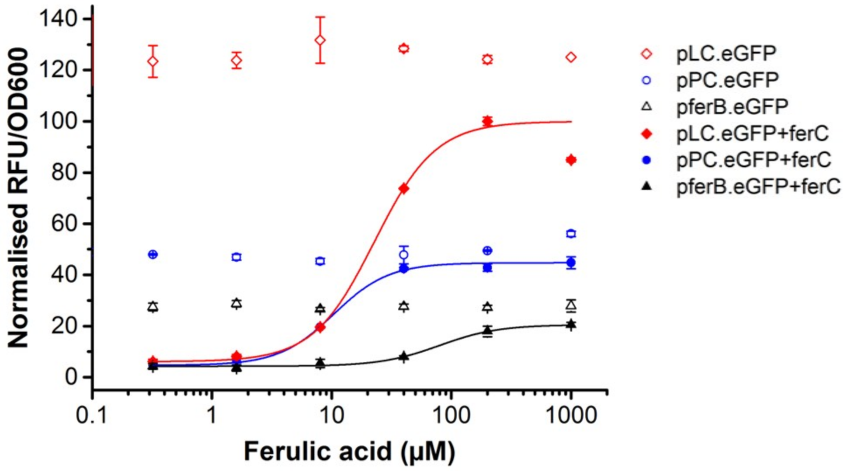

Figure S3: Reporters and Biosensor systems tested in E. coli DH10B. eGFP expression data in the absence (empty shapes) and presence of the *ferC* repressor (filled shapes), for the P_{ferB} (triangles), P_{PC} (circles), and P_{LC} (diamonds) biosensors in a *E. coli* K strain (DH10B). The fluorescent gene expression normalised to cell density (RFU/OD600) was expressed relative to the P_{LC} biosensor, and dose-response curves were fitted to increasing concentrations of ferulic acid.

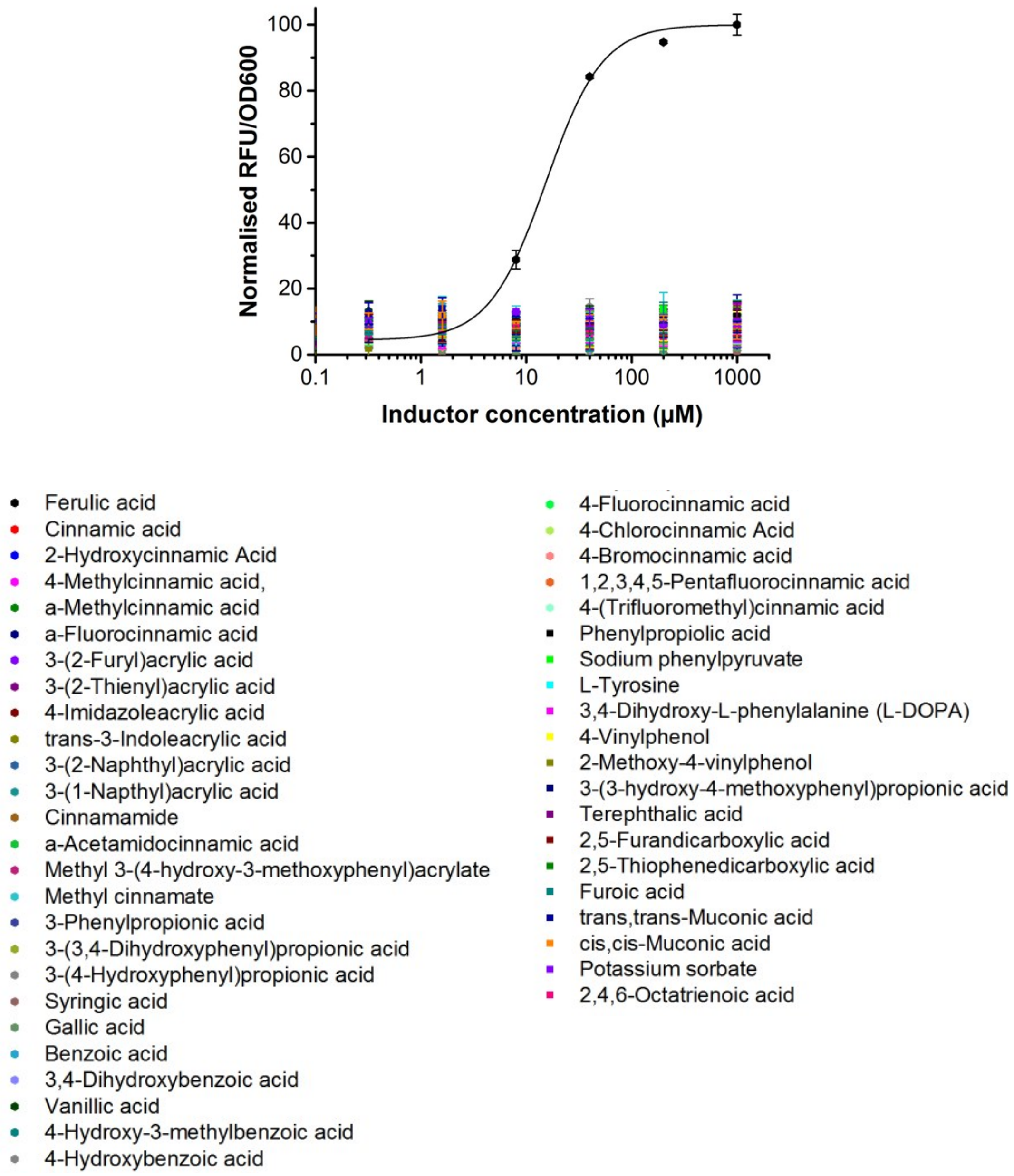

Figure S4: Biosensor non-responsive compounds. Basal gene expression for different compounds unable to activate the P_{LC} biosensor system and the dose response curve for ferulic acid. Fluorescence normalised to cell density (RFU/OD600) was expressed relative to the ferulic acid curve. Screening test was performed in *E. coli* BL21.

Table S1. Signal range (max/min) and fitted dose response curve data for the three biosensor systems in BL 21 and Top10F' strains.

Promoter	Strain	Signal range	Fitting Curve									
			Ebasal (RFU/OD)	error	Emax (RFU/OD)	p	error	EC50 (μM)	error	EC10 (μM)	EC90 (μM)	Sensing range
P_{LC}	BL 21	22.8	5.2	4.1	100.0	1.7	0.4	20.9	4.2	5.6	77.4	13.7
P_{PC}	BL 21	10.4	4.2	1.0	46.6	1.8	0.3	11.2	1.1	3.3	38.0	11.5
P_{ferB}	BL 21	5.0	3.7	0.1	17.0	1.9	0.1	60.5	2.3	19.2	190.8	9.9
P_{LC}	Top10F'	19.2	6.1	4.6	100.0	1.7	0.5	22.8	5.2	6.3	83.0	13.3
P_{PC}	Top10F'	12.4	4.7	0.7	44.7	1.9	0.3	10.4	0.7	3.3	33.3	10.2
P_{ferB}	Top10F'	4.7	4.3	0.3	20.5	1.8	0.2	79.6	7.5	22.9	276.2	12.0

Table S2. Signal range (max/min) and fitted dose response curve data for all responsive compounds tested.

	Tested compounds	Induction	Signal range	Fitting Curve									
				Ebasal (RFU/OD)	error	Emax (RFU/OD)	p	error	EC50 (μM)	error	EC10 (μM)	EC90 (μM)	Sensing range
**1**	Trans-Ferulic acid	High	26.2	4.4	1.26	100.0	1.6	0.11	15.3	0.9	3.9	59.5	15.1
**2**	p-Coumaric acid	High	25.0	2.6	2.80	100.0	1.2	0.17	26.1	3.8	3.9	174.1	44.6
**3**	3-(4-Aminophenyl)-2-propenoic acid	High	28.1	1.5	3.78	100.0	1.0	0.20	110.2	25.7	12.0	1008.8	83.8
**4**	Sinapic acid	High	15.4	2.3	2.49	100.0	0.8	0.12	314.4	55.8	19.8	4987.2	251.6
**5**	3-Hydroxy-4-methoxycinnamic acid	High	33.5	2.3	1.07	100.0	1.2	0.09	234.3	15.4	36.3	1513.9	41.7
**6**	3,4-Dihydroxy-5-methoxycinnamic acid	Moderate	14.8	2.9	1.85	100.0	0.7	0.10	746.5	117.7	33.9	16441.8	485.1
**7**	2,4-Dihydroxycinnamic acid	Moderate	9.6	6.1	0.79	100.0	1.2	0.09	823.4	45.6	131.2	5168.2	39.4
**8**	3,4-Dimethoxycinnamic acid	Moderate	11.4	4.7	0.28	100.0	1.7	0.06	825.0	13.2	227.6	2990.9	13.1
**9**	Caffeic acid	Moderate	11.2	3.9	0.98	100.0	1.3	0.18	1176.4	88.5	221.5	6247.9	28.2
**10**	4-Nitrocinnamic acid	Moderate	9.5	3.8	0.52	100.0	1.0	0.07	1564.5	96.8	157.9	15501.9	98.2
**11**	3,4,5-Trimethoxycinnamic acid	Low	6.7	4.6	0.44	100.0	1.2	0.14	2364.9	264.9	378.2	14786.7	39.1
**12**	3-(4-Hydroxy-3-methoxyphenyl) propionic acid	Low	3.3	5.7	0.31	100.0	1.2	0.19	4687.1	1189.6	701.8	31305.7	44.6
**13**	3-Methoxycinnamic acid	Low	4.8	3.9	1.02	100.0	0.9	0.48	9251.1	11084.4	815.7	104921.2	128.6

Table S3. CAS numbers and molecular structures of all compounds screened.

CAS number	Tested compounds		Molecular structure
537-98-4	**1**	**Trans-Ferulic acid**	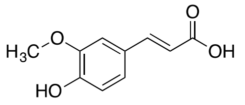
501-98-4	**2**	**p-Coumaric acid**	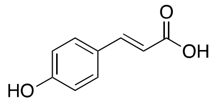
2393-18-2	**3**	**3-(4-Aminophenyl)-2-propenoic acid**	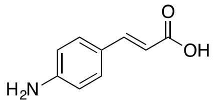
530-59-6	**4**	**Sinapic acid**	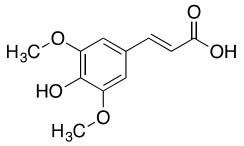
537-73-5	**5**	**3-Hydroxy-4-methoxycinnamic acid**	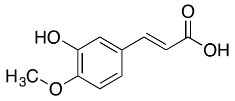
1782-55-4	**6**	**3,4-Dihydroxy-5-methoxycinnamic acid**	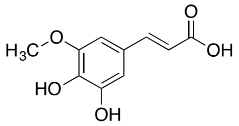
614-86-8	**7**	**2,4-Dihydroxycinnamic acid**	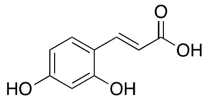
2316-26-9	**8**	**3,4-Dimethoxycinnamic acid**	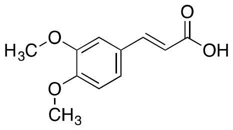
331-39-5	**9**	**Caffeic acid**	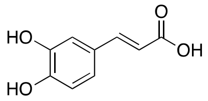
619-89-6	**10**	**4-Nitrocinnamic acid**	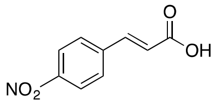
90-50-6	**11**	**3,4,5-Trimethoxycinnamic acid**	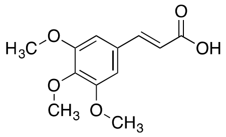
1135-23-5	**12**	**3-(4-Hydroxy-3-methoxyphenyl) propionic acid**	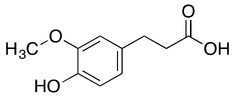
6099-04-3	**13**	**3-Methoxycinnamic acid**	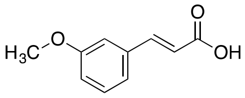

CAS number	Tested compounds		Molecular structure
140-10-3	**14**	**Cinnamic acid**	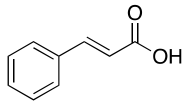
614-60-8	**15**	**2-Hydroxycinnamic acid**	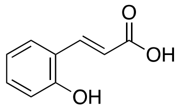
5469-45-4	**16**	**α-Acetamidocinnamic acid**	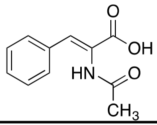
1199-77-5	**17**	**α-Methylcinnamic acid**	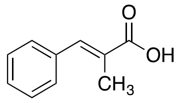
350-90-3	**18**	**α-Fluorocinnamic acid**	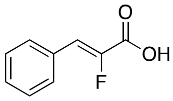
501-52-0	**19**	**Phenylpropiolic acid**	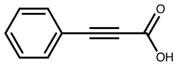
114-76-1	**20**	**Sodium phenylpyruvate**	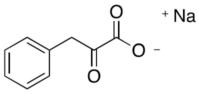
60-18-4	**21**	**L-Tyrosine**	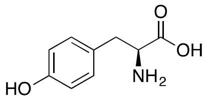
59-92-7	**22**	**3,4-Dihydroxy-L-phenylalanine (L-DOPA)**	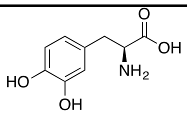
539-47-9	**23**	**3-(2-Furyl)acrylic acid**	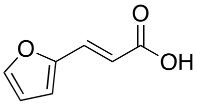
1124-65-8	**24**	**3-(2-Thienyl)acrylic acid**	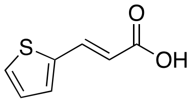
104-98-3	**25**	**4-Imidazoleacrylic acid**	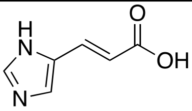
29953-71-7	**26**	**trans-3-Indoleacrylic acid**	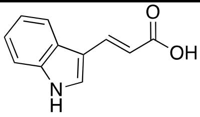
51557-26-7	**27**	**3-(2-Naphthyl)acrylic acid**	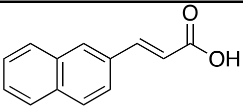
13026-12-5	**28**	**3-(1-Napthyl)acrylic acid**	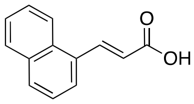

CAS number	Tested compounds		Molecular structure
1866-39-3	**29**	**4-Methylcinnamic acid**	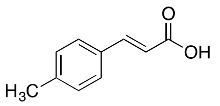
621-79-4	**30**	**Cinnamamide**	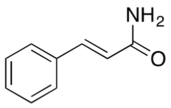
103-26-4	**31**	**Methyl cinnamate**	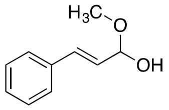
22329-76-6	**32**	**Methyl ferulate**	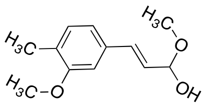
2628-17-3	**33**	**4-Vinylphenol**	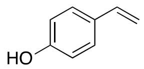
7786-61-0	**34**	**2-Methoxy-4-vinylphenol**	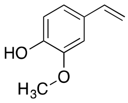
501-52-0	**35**	**3-Phenylpropionic acid**	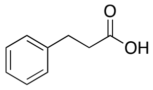
1078-61-1	**36**	**3-(3,4-Dihydroxyphenil)propionic acid**	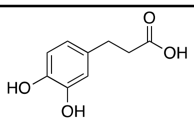
501-97-3	**37**	**3-(4-Hydroxyphenyl)propionic acid**	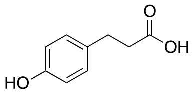
1135-15-5	**38**	**3-(3-hydroxy-4-methoxyphenyl)propionic acid**	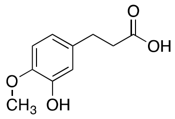
530-57-4	**39**	**Syringic acid**	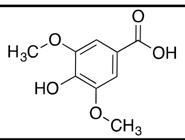
149-91-7	**40**	**Gallic acid**	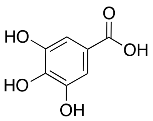
65-85-0	**41**	**Benzoic acid**	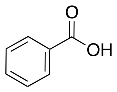
99-50-3	**42**	**3,4-Dihydroxybenzoic acid**	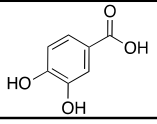
121-34-6	**43**	**Vanillic acid**	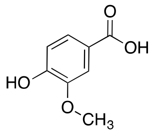

CAS number	Tested compounds		Molecular structure
499-76-3	**44**	**4-Hydroxy-3-methylbenzoic acid**	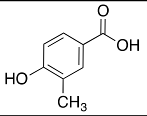
99-96-7	**45**	**4-Hydroxybenzoic acid**	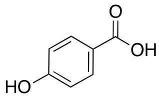
100-21-0	**46**	**Terephthalic acid**	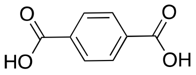
3238-40-2	**47**	**2,5-Furandicarboxylic acid**	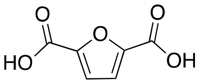
4282-31-9	**48**	**2,5-Thiophenedicarboxylic acid**	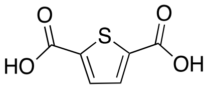
88-14-2	**49**	**Furoic acid**	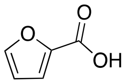
1615-02-7	**50**	**4-Chlorocinnamic Acid**	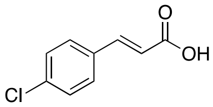
459-32-5	**51**	**4-Fluorocinnamic acid**	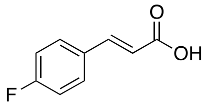
1200-07-3	**52**	**4-Bromocinnamic acid**	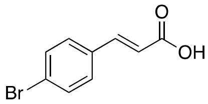
719-60-8	**53**	**1,2,3,4,5-Pentafluorocinnamic acid**	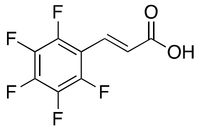
16642-92-5	**54**	**trans-4-(Trifluoromethyl)cinnamic acid**	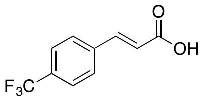
3588-17-8	**55**	**trans,trans-Muconic acid**	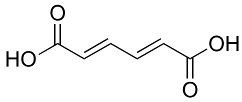
1119-72-8	**56**	**cis,cis-Muconic acid**	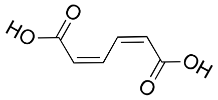
24634-61-5	**57**	**Potassium sorbate**	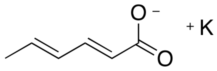
5205-32-3	**58**	**2,4,6-Octatrienoic acid**	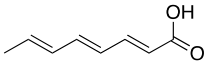

References

1 D. Kasai, N. Kamimura, K. Tani, S. Umeda, T. Abe, M. Fukuda and E. Masai, *FEMS Microbiol. Lett.*, 2012, **332**, 68–75.

2 R. Morra, J. Shankar, C. J. Robinson, S. Halliwell, L. Butler, M. Upton, S. Hay, J. Micklefield and N. Dixon, *Nucleic Acids Res.*, 2015.
